# Management Issues in Myasthenia Gravis Patients Living With HIV: A Case Series and Literature Review

**DOI:** 10.3389/fneur.2020.00775

**Published:** 2020-08-21

**Authors:** Jeannine M. Heckmann, Suzaan Marais

**Affiliations:** ^1^Neurology, Department of Medicine, Groote Schuur Hospital and University of Cape Town, Cape Town, South Africa; ^2^Neurology Research Group, UCT Neuroscience Institute, University of Cape Town, Cape Town, South Africa

**Keywords:** HIV, myasthenia gravis, autoimmune, immune restoration, immunosuppressive therapy, rituximab, methotrexate

## Abstract

South Africa is home to more than seven million people living with human immunodeficiency virus (HIV) and a high prevalence of tuberculosis. Human immunodeficiency virus–infected individuals may develop myasthenia gravis (MG), which raises questions regarding their management. An MG database, with 24 years of observational data, was audited for HIV-infected persons. Case reports of MG in HIV-infected persons were reviewed. We identified 17 persons with MG and HIV infection. All had generalized MG with a mean age at onset of 37.8 years. Eleven had acetylcholine receptor antibody–positive MG; one had antibodies against muscle-specific kinase. Six developed MG prior to HIV infection (mean CD4^+^ 361 cells/mm^3^); four worsened <6 months of starting antiretrovirals. Eleven developed MG while HIV-infected (mean CD4^+^ 423 cells/mm^3^); five presented with mild MG; three in MG crisis requiring rescue therapies (intravenous immune globulin or plasma exchange and/or intravenous cyclophosphamide). Two were diagnosed with HIV infection and MG at the same time. Fifteen required maintenance steroid-sparing immune therapies, predominantly azathioprine, or methotrexate. Plasma HIV viral loads remained below detectable levels on antiretrovirals during immunosuppressant treatment. Over the average follow-up of 6 years, 10 achieved minimal manifestation status, and the remainder improved to mild symptoms. Three cases had tuberculosis before MG, but none developed tuberculosis reactivation on immunosuppressive therapy; one used isoniazid prophylaxis. Herpes zoster reactivation during treatment occurred in one. Conclusions include the following: MG in HIV-infected patients should be managed similarly to individuals without HIV infection; half develop moderate–severe MG; MG symptoms may worsen within 6 months of antiretroviral initiation; safety monitoring must include plasma HIV viral load estimation. Isoniazid prophylaxis may not be indicated in all cases.

## Introduction

Myasthenia gravis (MG) has a similar incidence worldwide (1). However, South Africa is also home to more than seven million people living with human immunodeficiency virus (HIV), and therefore the co-occurrence of MG in some persons living with HIV infection is expected. In the early 2000's, South Africa rolled out the largest governmental-sponsored antiretroviral treatment (ART) program globally. Initially, ART triple therapy was provided only to those with CD4^+^ count of <200 cells/mm^3^, but since 2016, ART has been available to all with HIV infection regardless of CD4^+^ count. Present first-line ART comprises efavirenz, tenofovir (TDF), and emtricitabine. South Africa provides treatment to ≈4.4 million HIV-infected people (2).

In Africa, HIV spreads predominantly through heterosexual transmission, and hepatitis B (HepB) coinfection is rare, but tuberculosis is common (3). As HIV-infected people are at risk of opportunistic infections, and this risk may be increased with comorbid autoimmune diseases requiring immunosuppressive therapies, we audited the results of our HIV-infected patients with MG. We have summarized our results with reported cases to develop empiric management guidelines.

## Materials and Methods

The diagnosis of MG was based on clinical criteria of fatigable weakness and responsivity to anticholinesterases, repetitive nerve stimulation (RNS), and/or acetylcholine receptor (AChR)–antibody (ab) testing as previously reported (4, 5). Although routine muscle-specific kinase (MuSK)-ab testing is unavailable, AChR-ab–negative sera were tested for MuSK-abs between 2006 and 2015 (6). Observational data have been collected using standardized forms since 1997. Data captured MG Foundation of America (MGFA) disease outcomes (7), MG composite scores (8), drug dosages, and complications thereof, hospitalization events, and opportunistic infections. The registry (R004/2014) and audit (HREC 611/2013) were approved by the institutional ethics committee.

Although screening for HIV was not routinely performed before 2012, since then HIV, HepB, and HepC infection screening occurred prior to starting immunosuppression.

To review the literature, PubMed was searched for articles published in English (1997–2019) with the terms “HIV” or “AIDS” and “myasthenia gravis” and from manual searching reference lists.

## Results

Seventeen patients were identified in the MG database (*n* = 844 entries) who were also living with HIV (2003–2019); six were diagnosed with MG and subsequently became HIV-infected; nine were HIV-infected on effective ART [viral load (VL) <20 copies/ml or lower than detectable level (LDL)] prior to developing MG; and two were diagnosed with HIV and MG at the same time (Table 1).

**Table 1:** Clinical characteristics of patients with concomitant MG and HIV infection.

	**MG-HIV** ** (*n* = 6)**	**HIV-MG** ** (*n* = 11)**	**Case reports 1998–2019 (*n* = 13)**
Sex, female, *n* (%)	6 (100)	8 (73)	7 (54)
Age at MG symptom onset, mean ±*SD*, years	30.8 ± 14.9^*^	39.6 ± 8.6^*^	38.2 ± 18.6
CD4^+^ count, mean ±*SD*, cells/mm^3^	361 ± 133^#^	423 ± 76^#^	428 ± 315
**Diagnostic Criteria**, ***n*** **(%)**			
AChR ab^+^	6 (100)	5 (45)	4 (31)
MuSK ab^+^		1 (9)	4
AChR ab^−^/RNS^+^		4 (36)	5 (38)
AChR ab^−^/RNS^−^/CHEI^+^		1 (9)	0
**MGFA Grade Nadir**, ***n*** **(%)**			
2a/b	2 (33)	5 (45)	9 (69)^⋎^
3b	1 (17)	3 (27)	
4b/5	3 (50)	3 (27)	
Concomitant autoimmune disease		2 (PM/IBM, ATD)	0
**MG treatments, average doses when HIV**^**+**^			
Prednisone (max doses), (*n*) mg/kg	0.14 (1)	0.52 ± 0.3 (10)	(6)
Azathioprine, mean ±*SD* (*n*), mg/kg	1.2 ± 0.1 (4)^##^	2.1 ± 0.3 (5)^##^	(2)
Methotrexate, weekly, mean ±*SD* (*n*), mg		15.6 (4)	
Mycophenolate mofetil (*n*)		2 × 1,250 mg (1)	(1)
Cyclosporine (*n*)		2 × 150 mg (1)^α^	(1)
Cyclophosphamide pulses, (*n*)		5 × 250 mg (1)	
Rituximab cycles (*n*)		4+2 (1)	(1)
IVIG/Plasma exchange, *n* (%)		3^β^/1^χ^ (4)	(5/2)
MG crises after MG diagnosis/treatment in HIV^+^	0	1	1
Minimal manifestation status, *n* (%)	4 (80)	6 (55)	UK
Patients on continued IS therapy, *n* (%)	3 (60)	10 (91)	UK
Follow-up since comorbid MG/HIV diagnosis, mean ±*SD* (*n*), years	11.8 ± 5.2^**^	3.9 ± 3.1^**^	1.2 ± 0.8 (12)
HIV viral load <20 copies on follow-up	6 (100)	11 (100)	UK

*MG-HIV refers to the patients with MG who later became infected with HIV; HIV-MG refers to patients living with HIV who later manifested MG. Four HIV-MG cases developed MG ≥45 years. No cases had thymoma-MG*.

* *Refers to MG diagnosis before the patient became HIV-infected vs. HIV-MG (p = 0.14)*.

** *p = 0.009*.

# *p = 0.53*.

## *p = 0.033*.

*ATD, autoimmune thyroid disease; PM/IBM, polymyositis/inclusion body myositis overlap; HIV^+^, known to be HIV-infected; AChR ab^+^, acetylcholine receptor ab–positive; MuSK ab^+^, muscle-specific kinase ab^+^; AChR ab^−^, not tested for MusK-abs; CHEI^+^, responsivity to cholinesterases; IVIg, intravenous immunoglobulin; RNS^+^, 3-Hz repetitive nerve stimulation >10% decrement; IS, immunosuppressive*.

α *3 months before renal dysfunction*.

β *Three of the IVIg course were administered at MG diagnosis in crisis (MGFA grade 5)*.

**Figure 1:**
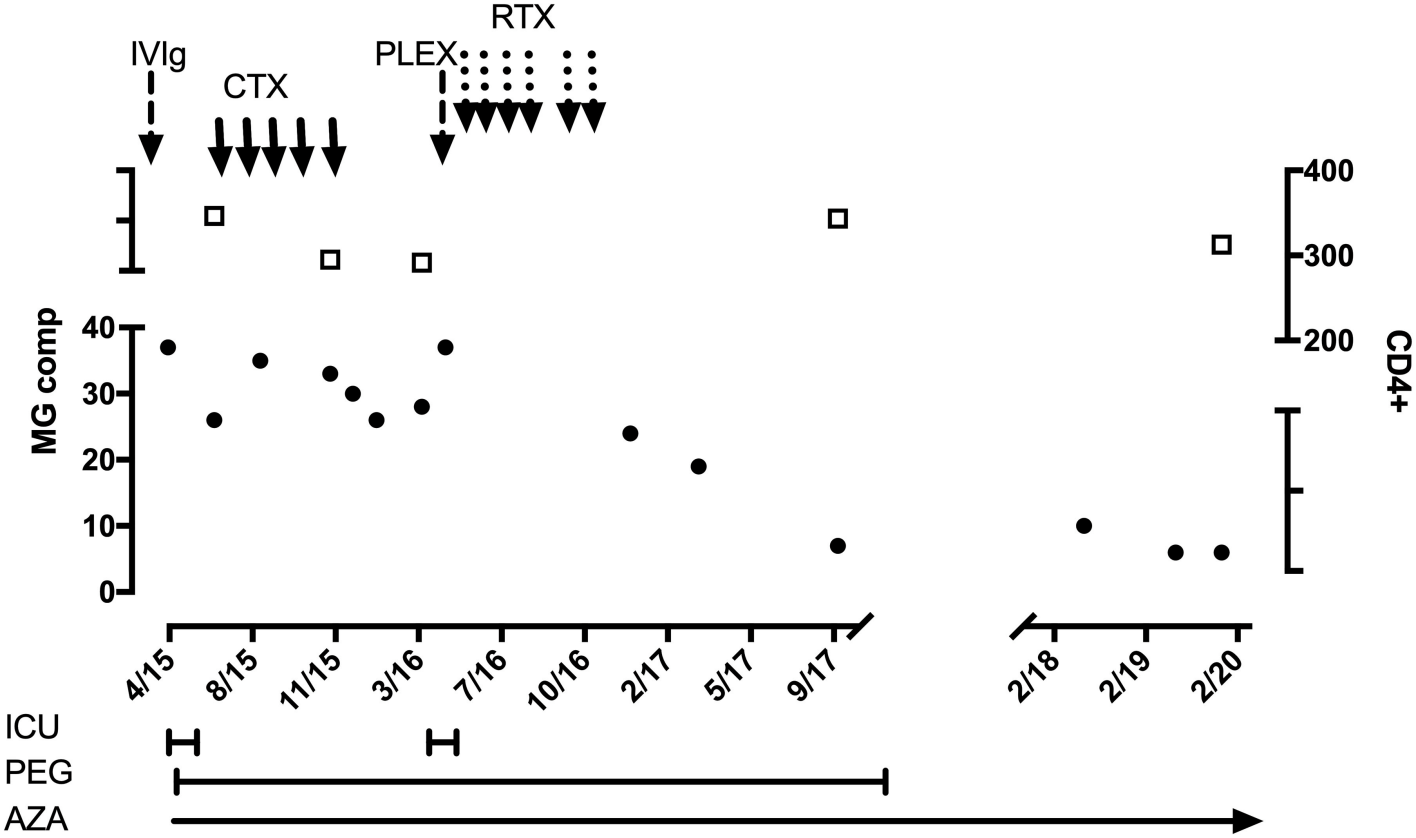
HIV-infected patient with MuSK-MG. Black circles and left-sided *y* axis refers to MG composite score. Open squares and right-sided *y* axis refers to CD4^+^ count. ICU, intensive care unit; PEG, percutaneous endoscopic gastrostomy; AZA, azathioprine; IVIg, hyperimmune intravenous globulin; CTX, cyclophosphamide monthly pulses (250 mg); PLEX, plasma exchange; RTX, rituximab infusions (500 mg weekly × 4; and 2 weekly × 2 after 3 months).

χ *Plasma exchange during MG relapse months after diagnosis (Figure 1). Rituximab cycles: 375 mg/m^2^ 2 weekly × 2, monthly × 2, and then at 6 months*.

⋎ *MGFA grade 2a/b assigned to descriptions of mild disease. Rituximab cycles: 375 mg/m^2^ 4 weekly × 2, monthly × 2*.

*Case reports (1998–2019) (9–20)*.

### MG Patients on Immunosuppressant Therapy Becoming Infected With HIV (MG-HIV Group)

Six women with AChR-ab–positive MG were diagnosed with HIV infection between 1.5 and 40 years after developing MG. Four had thymectomies <3 years of symptom onset. All were receiving immunosuppression at the time of presumed seroconversion: five had reached MGFA minimal manifestation status (MMS), and one had mild symptoms (grade 2A).

Three developed skin rashes, which prompted HIV testing between 1, 2, and 10 years after MG diagnosis. One patient developed a flulike illness 12 years after azathioprine initiation, which was followed by a declining leukocyte count on routine monitoring when HIV infection was confirmed. After stopping azathioprine, the CD4^+^ count rapidly increased from <100 to >500 cells/mm^3^. She remained off all immune therapy for 9 years requiring only pyridostigmine for MG symptoms. Antiretroviral treatment was started when the CD4^+^ count declined to ~200 cells/mm^3^. Myasthenia gravis symptoms subsequently worsened over several months, and 12 months after ART initiation, a lower dose of azathioprine was reinitiated (CD4^+^ ~250 cells/mm^3^), resulting in symptomatic improvement. Another patient developed unexplained weight loss of 20 kg, which prompted HIV testing (CD4^+^ ~154 cells/mm^3^). As she was asymptomatic, azathioprine was discontinued, and the CD4^+^ count recovered to ~650 cells/mm^3^. Within 6 months, azathioprine was reinitiated, at a lower dose, because of recurring bulbar symptoms. The remaining patient's MG was in remission, and her immunotherapy was being weaned when she tested HIV-positive.

These patients have been followed up for an average of 11.8 years since their HIV diagnosis. Four remain on azathioprine, although the doses required to control their disease before HIV infection was detected were significantly higher compared to the doses required to control MG after ART was reintroduced (2.6 mg/kg; *SD* ±0.1 vs. 1.2 ± 0.1; *p* < 0.0001). Two patients were in MG-MMS and were weaned off azathioprine when testing positive for HIV infection; one has remained in remission for 14 years, but the other developed bulbar symptoms after 10 years and was reinitiated on azathioprine (VL-LDL). The two patients without thymectomies were weaned off prednisone maintaining MMS on maintenance treatment. One patient had pulmonary tuberculosis on two occasions, more than 3 years prior to MG, but was not started on isoniazid prophylaxis when ART was commenced.

### Patients Living With HIV Who Subsequently Developed MG (HIV-MG Group)

Eleven HIV-infected people developed MG. Nine were receiving ART [mean, 5 years (*SD*, ±3.9)] prior to developing MG symptoms, whereas two tested HIV-positive at the time of MG diagnosis. The mean CD4^+^ count at MG diagnosis was 423 cells/mm^3^, although three, who had been on effective ART (VL-LDL) had CD4^+^ counts of <200 cells/mm^3^ (range, 173–190 cells/mm^3^).

Three patients presented in MG crisis after 6–12 months of symptoms. Three had a history of tuberculosis 2–11 years before manifesting with MG, but none developed reactivation of tuberculosis on immunosuppressive therapy; isoniazid prophylaxis was used in one case. One individual developed herpes zoster reactivation during MG treatment.

#### Cases Who Were Concurrently Diagnosed With HIV and MG

A patient was diagnosed with HIV infection (CD4^+^ ~160 cells/mm^3^) when admitted in MG crisis following symptoms for 12 months. Acetylcholine receptor antibody testing was negative (MuSK-abs not tested), but with decremental RNS. She required ventilation, but responded rapidly to intravenous immunoglobulin (IVIg) and prednisone (0.8 mg/kg). She was initiated on ART within 1 week and a month later on azathioprine (1.4 mg/kg). At 12 months, she was asymptomatic, and ART was effective (VL-LDL).

Another case presented with generalized MG (grade 3B), which developed over 4 weeks. Acetylcholine receptor antibody testing was negative (MuSK-abs not tested), but RNS showed a decremental response, and he responded to pyridostigmine. He was found to be infected with HIV (CD4^+^ ~700 cells/mm^3^) and HepB (HepC-negative). Antiretroviral treatment and prednisone were initiated, and he improved so rapidly that steroid-sparing therapy was omitted. After 6 months on ART (VL-LDL), he was only mildly symptomatic, and prednisone was successfully weaned over several months.

#### Cases Living With HIV Infection Developing MG

The ages of these patients ranged between 28 and 53 years. Five cases had circulating AChR-abs, one had MuSk-abs, and three were AChR-ab–negative (MuSK-abs not tested) but responded to anticholinesterases.

One case who had been virally suppressed on ART for 5 years developed a detectable VL as a result of an inability to swallow the ART tablets. Three months later, she was diagnosed with MG grade 3B and was initiated on pyridostigmine, increasing prednisone doses (0.9 mg/kg) and azathioprine (1.9 mg/kg). At 12 months, the MG was in MMS, and ART was effective (VL-LDL).

The drug dosages in newly diagnosed HIV-MG cases were similar to those who were diagnosed with MG and became HIV-infected years later [azathioprine 2.1 vs. 2.6 mg/kg in MG (pre-HIV); *p* = 0.12]. Four cases were treated with weekly methotrexate (range, 10–20 mg) and one with mycophenolate mofetil 2,500 mg daily for 5 years. The average follow-up since MG diagnosis has been 3.9 years (range, 0.5–10 years). Five achieved persistent MMS, one without treatment, and the other improved to mild MG on maintenance therapy, and therefore none with AChR-abs underwent thymectomy.

### Special Case Scenarios

#### MuSK-MG

This woman with severe oculobulbar MG manifesting over 6 months was reported previously (6). She had received effective ART for 4 years. She was admitted in myasthenic crisis (Figure 1) and showed a transient response to pyridostigmine and IVIg. However, she developed steroid-induced psychosis resulting in her refusing plasma exchange. Instead, 5 monthly cyclophosphamide infusions [one-third of 500 mg/mm^2^ (21)] were administered together with azathioprine and isoniazid prophylaxis. During this time, she had improved slowly, until she relapsed into MG crisis precipitated by pneumonia. She agreed to plasma exchange, which was followed by rituximab infusions (375 mg/mm^2^) and a steady recovery. She currently remains asymptomatic on azathioprine and ART. Interestingly, within 6 months of starting azathioprine (2.3 mg/kg), her γ-glutamyl transferase (GGT) increased to 3× the upper limit of normal, and isoniazid was discontinued. Subsequently, hepatic transaminases (aspartate transaminase and alanine transaminase) and GGT increased to 4× the upper limit, which normalized after an azathioprine dose reduction (1.1 mg/kg). During the stormy course of MG requiring cyclophosphamide and rituximab infusions, leukocytes remained >3 × 10^9^/L, polymorphs >2 × 10^9^/L, lymphocytes ≥0.7 × 10^9^/L, CD4^+^ ~222/mm^3^, and VL-LDL.

#### MG With HIV Inflammatory Myopathy/Inclusion Body Myositis

This middle-aged woman developed proximal weakness over several years since HIV infection was diagnosed and ART initiated. She presented to the neurology service after developing, over 2 years, additional symptoms of fatigable diplopia and bulbar symptoms accompanied by limb fatigability. At presentation, she was receiving effective ART, and serum creatine kinase was raised (1.5× upper limit). Although the serum AChR-abs (and muscle autoimmune panel) were negative, MG was confirmed by a decremental response on RNS and responsivity to intramuscular neostigmine (swallowing and leg strength). A muscle biopsy showed endomysial fibrosis and fiber size variation, but no rimmed vacuoles. Treatment for MG was started viz. pyridostigmine, steroids, and methotrexate. The MGC score improved by 50% over 12 months, but she developed moderate weakness of hand flexors and knee extensors. The prednisone has been weaned, and she remains on methotrexate 20 mg weekly. The working diagnoses include MG, which is at present minimally symptomatic, and HIV-associated inflammatory myopathy overlapping with inclusion body myopathy (22). The current goal is to wean the methotrexate to the lowest dose maintaining control of MG.

#### Drug–Drug Interactions

Another case was diagnosed with AChR-ab–positive MG (grade 2B) after 3 months of symptoms. She had been on effective ART for 4 years (CD4^+^ 200 cells/mm^3^; VL-LDL) and had been treated for tuberculosis twice, at least 2 years prior to the diagnosis of HIV and the onset of MG symptoms. Prednisone and methotrexate were started together with isoniazid prophylaxis for 18 months because of associated bronchiectasis. Her CD4^+^ count remained stable (VL-LDL), but deteriorating bulbar MG symptoms required high-dose prednisone. After 12 months, methotrexate was replaced with cyclosporine, and although the MG responded within 4 months, her kidney function deteriorated (rising urea/creatinine). The ART regimen contained TDF, which was replaced with zidovudine. The cyclosporine dose was initially reduced, but eventually replaced with azathioprine. Although the renal function improved, she developed aplastic anemia. The ART regimen was then altered to include abacavir, nevirapine, and lamivudine, and given that she had achieved MMS, the azathioprine was discontinued. Within weeks, her bulbar symptoms recurred, and she was cautiously started on mycophenolate mofetil (MMF) (500 mg daily) with prednisone. The MMF was increased to 2,500 mg daily. After 15 months, she started improving, and the prednisone dosing could be reduced by 50%. Four years later, she remains in remission on MMF.

### Literature Review

Thirteen case reports were identified and summarized in Table 1 (see legend). One patient had MG-HIV, two were diagnosed with HIV when they presented with mild MG (CD4^+^ 250–350 cells/mm^3^), and 10 had HIV-MG. More than 80% had mild–moderate MG symptoms. Only one case presented with MG and a CD4 count of <200 (CD4^+^ 63 cells/mm^3^). At least five were on effective ART (VL-LDL) when they developed MG. Five showed mild symptomatic deterioration 3 weeks to 3 months after ART initiation or adjustment (for improved efficacy).

## Discussion

We describe a cohort of HIV-infected MG patients, followed for many years, unlike previous cases reports with <2 years follow-up. Several findings need highlighting. Most cases known with HIV infection develop MG with relatively preserved CD4^+^ counts and/or mild disease at onset. However, HIV-MG may present in crisis and with lower CD4^+^ counts [ ≤ 222 cells/mm^3^ in three cases, and (23)].

### Immunomodulatory Therapy in HIV-Infected MG Cases

Four HIV-MG patients required intensive and multiple immune therapies to gain control of their MG despite ART, which included “rescue” therapy with plasma exchanges/IVIg and/or induction therapy with cyclophosphamide or rituximab in addition to maintenance therapies (prednisone and steroid-sparers). Methotrexate is a cost-effective alternative in generalized MG (24), and four HIV-infected people, who were not potentially child-bearing, were managed with methotrexate, and two obtained MG remission status within 6 months.

In addition to our case, one other reported HIV-infected MuSK-MG patient was treated with rituximab. In both, the protocols comprised 4 weekly, followed shortly after by 2 weekly infusions. Jing et al. (25, 26) and our experience (unpublished) in HIV-uninfected patients have found excellent responses after a single ≈500–600 mg rituximab dose, which may last for 9 to more than 42 months; this regimen should be explored in HIV-infected cases with MG.

In total, 16 of 17 patients received prednisone and steroid-sparing immunomodulatory therapies. With the exception of one patient who developed a herpes zoster reactivation rash, none developed opportunistic infections during such treatment. It should be noted that all patients receiving ART remained virally suppressed on immunomodulatory therapy. Maintaining effective ART (VL-LDL) while taking immunosuppressive therapies is critically important. New guidelines advise that if the CD4^+^ count was >200 cells/mm^3^ before starting immunosuppressive therapies, monitoring of the HIV-VL is the most cost-effective (27). Despite effective ART, MG symptoms may recur years later. It is prudent to then consider additional autoimmune thyroid disease, hormonal-related fluctuations (pregnancy, menopause), and drug–drug interactions. Overall, HIV-infected patients with MG should be managed similarly to HIV-uninfected cases: immunomodulatory therapies should be administered according to the severity of MG.

Although it was shown that thymectomy in AChR-ab generalized MG resulted in lower prednisone doses required to improve MG and maintained for 5 years (28), it was not performed here mainly because these patients appeared to improve and maintain MMS with successful weaning of prednisone.

### Effects of Level of Immunosuppression and Immune Recovery on MG

Two main groups were encountered: MG patients who were well-controlled on immunosuppressive therapy when they became infected with HIV and could subsequently be managed on lower doses of azathioprine compared to pre-HIV dosing (1.3 vs. 2.6 mg/kg; *p* < 0.0001) and patients who developed MG after HIV acquisition and who required similar doses of immunotherapies to that used in HIV-uninfected populations (29) (2.1 vs. 2.6 mg/kg, respectively; *p* = 0.12).

Antiretroviral treatment initiation within the first 3 months may be associated with a subclinical “cytokine storm” (30), and recovery of the immune system may take many months as shown by CD4^+^ count recovery (31). During this period, MG symptoms may deteriorate as was evident from four MG-HIV cases who were weaned off immunosuppressants when diagnosed with HIV, but had to be reinitiated within 6 months of starting ART, albeit with lower doses. A previous report (19) described an HIV-infected man (CD4^+^ 290 cells/mm^3^) who developed bulbar MG shortly after the addition of ritonavir to his two-drug ART regimen. The authors suggested that MG occurred as a side effect from ritonavir. At present, in South Africa, it is estimated that there are 200,000 patients on second-line ART (2), which encompasses protease inhibitors (ritonavir). One of our cases received ritonavir without MG deterioration. Our experience with this class has shown few neuromuscular side effects (32). It is more likely that the reported patient (19) worsened due to immune “recalibration” with more effective ART.

Isolated cases presenting with MG at the same time as HIV infection have improved alongside ART with or without a short course of prednisone in addition to anticholinesterases and not requiring long-term immune therapy.

### Investigations Prior to Immunomodulatory Therapy

Coinfection with HepB/HepC must be excluded. Baseline blood laboratory values should be determined prior to starting or adding immune therapies to the medication list so that drug-induced complications are easily identifiable. If an expected drug-associated side effect occurs, such as raised transaminases with azathioprine, a lower dose may be all that is required to safely continue the drug (33). Prior to rituximab, screening for previous HepB infection (anti-HepB surface-ab^−^ and anti-HepB core-ab–positive, but HepB surface-antigen negative) must be performed because 25–40% of these cases may seroconvert to active HepB after rituximab (34). The WHO estimates that only 10% of HepB-infected people are aware of their infection status (who.int/news-room/fact-sheets/detail/hepatitis-b).

It is important to screen for active tuberculosis prior to starting immunosuppressive therapy. Among HIV-infected people, cases at particular high risk include those with prior tuberculosis exposure and/or diabetes (35). Screening should include a chest X-ray to exclude active tuberculosis or identify evidence of fibrotic scarring. Human immunodeficiency virus–infected people have 3–20 times higher risk of reactivation of latent tuberculosis compared to the general population (36). In resource-rich areas with low background prevalence of tuberculosis, diagnostic tests, such as the interferon γ release assays (QuantiFERON-TB Gold Plus; T-SPOT test), can be useful to identify infected individuals. However, the results have to be interpreted with caution in areas with high tuberculosis prevalence. These tests may be falsely negative after starting immune therapies (37). False-positive tests may be a response to BCG-vaccinations, environmental exposure to non-tuberculous mycobacteria (35), and in autoimmune diseases (37).

### Prophylactic Treatment for Tuberculosis During Immunomodulatory Therapy

While some have suggested using prophylactic antifungal and antituberculosis therapies in patients on immunosuppressive therapy (38), we do not follow this practice routinely, although we screen for active tuberculosis by questionnaire and chest radiograph. We recommend that, in tuberculosis-endemic areas, each patient's comorbidities be considered for possible isoniazid preventive therapy. With fibrotic lung lesions, isoniazid preventive therapy for 6–12 months reduced the odds for reactivation of latent tuberculosis by ≈50% (35), but must be given with 25 mg pyridoxine (39).

The conclusions that can be drawn from these cases include the following: MG in HIV-infected people should be managed similarly to HIV-uninfected individuals; transient worsening of MG may occur weeks to months following ART initiation; monitoring should comprise HIV VLs estimation; and isoniazid prophylaxis is not indicated in all cases.

## Data Availability Statement

All datasets generated for this study are included in the article/supplementary material.

## Ethics Statement

The studies involving human participants were reviewed and approved by University of Cape Town Human Research Ethics Committee. Written informed consent for participation was not required for this study in accordance with the national legislation and the institutional requirements.

## Author Contributions

JH performed the case, literature review, and drafted the manuscript. SM edited the manuscript drafts and contributed to the literature review. All authors contributed to the article and approved the submitted version.

## Conflict of Interest

The authors declare that the research was conducted in the absence of any commercial or financial relationships that could be construed as a potential conflict of interest.
